# IMPROVING ACCESS AND OUTCOMES FOR RURAL CAREGIVERS USING THE TELEPHONE

**DOI:** 10.1093/geroni/igz038.2265

**Published:** 2019-11-08

**Authors:** Linda Nichols, Jennifer Martindale-Adams

**Affiliations:** 1 VA Medical Center, Memphis, Tennessee, United States; 2 Department of Preventive Medicine, Memphis, Tennessee, United States

## Abstract

Many caregivers of older rural veterans have limited access to services. In 2017, VA’s Office of Rural Health and Caregiver Support Program funded the Memphis Caregiver Center to deliver Resources for Enhancing All Caregivers Health (REACH VA) as a national behavioral intervention to improve access to care. To date, 438 caregivers of older Veterans have enrolled. REACH is a proven 4-session caregiver intervention, structured and easily replicated. Rural internet service is often problematic. Offering REACH by telephone provides many benefits, including access to service not available in the rural area, almost universal technology penetration, ease and familiarity of use, and removal of barriers of travel and care for the loved one. Through this enhanced service, caregivers of older veterans with dementia, PTSD, ALS, MS, and other conditions have shown statistically and clinically significant improvements in managing concerns and safety relating to their loved one and their own stress and coping.

